# The Poor-Law Infirmary

**Published:** 1913-03-08

**Authors:** 


### THE POOR-LAW INFIRMARY.

### I.—The Present Position of the Infirmary Doctor.

At present our Poor-Law infirmaries occupy a somewhat anomalous position. They are not acknowledged as teaching schools, yet, as is well known, they possess some of the finest material for clinical teaching that can be found. They have on their staffs men who are expert surgeons and physicians—men of experience and ability whose skill and reputation are well known to their professional colleagues. Every year they recruit a small army of young practitioners who find in the work an onerous but highly useful introduction to the routine of general practice. Some of these recruits stay on the staff; they become infirmary men, interesting themselves in their institutional work just as much as the members of hospital staffs interest themselves in their work. Others, and perhaps the majority, vanish from the infirmary world into general or special practice. But all agree that the experience derived from such work is useful and valuable.

It has often been suggested that the medical student should become acquainted with the work that is carried on in these institutions, and that he should familiarise himself with the class of patient who nowadays goes not to the general hospital but to the infirmary. Usually it is presumed that these cases are the chronic everyday sort of diseases. As a matter of fact some of the most interesting cases, professionally and scientifically, are warded in infirmaries and not in the general hospitals—perhaps an added inducement to throw open the infirmary doors to the student of medicine. We shall discuss in future articles this relation of the infirmary to the student and the teaching of medicine; for the moment we merely refer to it to point out one of the subjects on which we should like from our readers who are in a position to criticise and to judge an expression of opinion.

No one who seriously studies the position of hospitals at the present moment can, we think, have much doubt that the infirmary will considerably extend in influence, importance, and prestige in the near future. The introduction of National Insurance, with the many problems it raises, will doubtless have an effect upon these institutions—an effect which at the present time it is exceedingly difficult to gauge. While much remains to be done, it is also a fact that during the last decade our infirmaries have considerably improved in every way. They can no longer be all tarred with the old brush of inefficiency; we must discriminate between them and admit that, while there are still institutions which are a disgrace to the community, there are many more in whose existence we must justifiably take a pride. The improvement has been all along the line; in structure, administration, treatment, nursing, and general working there has been betterment to which the unprejudiced observer cannot shut his eyes. That being the case, it becomes time for the public to recast its notion with regard to the scope and nature of the work that the infirmary executes. Hitherto much of this work has been done in the dark; even the ratepayers who support the institutions have known next to nothing of what goes on in them. The glare of publicity that is so constantly and effectively focussed upon our voluntary hospitals by the press and by annual reports hardly ever turns upon the Poor-Law institutions, with the result that the public is in profound ignorance of what an infirmary means, what it accomplishes, and what it may further accomplish provided it receives proper support and is efficiently controlled and managed.

It is the fashion to draw a comparison between the infirmary and the voluntary hospital, to the disfavour of the former. In our opinion this comparison is unfair to both institutions alike. They have a different scope and aim, and their methods of working are essentially different, while the class of patient for whom they cater is equally different. No fair comparison can therefore in the nature of things exist, until, at least, these two classes of institutions approach each other more closely than they do at present. In dealing with the infirmary we must have different standards from those we employ in dealing with the voluntary hospital. We do not for a moment mean to imply that by this we assert that these differences are differences in efficiency. We have always maintained that a hospital, whether Poor-Law or voluntary, to be really a good hospital must be efficient—that is to say, it must be modern, up-to-date, and as perfect as it can be made under existing circumstances. The supreme test, after all, must be the test of utility, and that test can and must be applied to both voluntary hospital and Poor-Law infirmary.

In both cases certain allowances must be made. The Poor-Law hospital is not worried by financial difficulties. The voluntary hospital has a chance of selecting patients which the infirmary lacks. The work of the infirmary is often carried out under most adverse conditions; that of the large general hospital usually under ideal or almost ideal conditions. Obviously, then, the standards to be applied in testing the ultimate usefulness of the two institutions must differ, however much we may insist on the primary consideration of efficiency. The public and the profession must be made to realise this, and to give the infirmary its due share of credit in order to judge, finally, what reforms and improvements are needed and how these reforms are to be carried out.

We hope in this series of articles to approach the subject of infirmary interests from all points of view. Especially do we hope that infirmary workers through our columns will give each other the benefit of their experience and knowledge, and share, in short, their internal institutional tips of proved success. We shall welcome articles on infirmary subjects, which, in accordance with our rule, will be paid for if accepted and published. There are many such subjects, and we feel sure that there are many workers able and willing to write about them, to give their views clearly, and to point out in what direction improvement is called for. A full discussion is for everyone's good by informing others, who do not know the facts, of the existing state of things inside other institutions. We therefore throw out this appeal, convinced that infirmary workers will find such a series of articles as The Hospital has initiated helpful to them, and convinced also that they will aid us to make it more helpful by their co-operation and assistance. For a journal is just as good as its readers choose to make it.

